# Hypertension Status Moderated the Relationship between the Hippocampal Subregion of the Left GC-ML-DG and Cognitive Performance in Subjective Cognitive Decline

**DOI:** 10.1155/2022/7938001

**Published:** 2022-10-15

**Authors:** Guiyan Cai, Yajun Wang, Ruilin Chen, Shurui Xu, Jia Luo, Qianqian Sun, Ming Li, Hui Lin, Qinyuan Zhang, Yimei Zheng, Hanni Lin, Jiao Liu

**Affiliations:** ^1^College of Rehabilitation Medicine, Fujian University of Traditional Chinese Medicine, Fuzhou, Fujian 350122, China; ^2^Affiliated Rehabilitation Hospital, Fujian University of Traditional Chinese Medicine, Fuzhou, Fujian 350122, China; ^3^Physical Education Department, Fujian University of Traditional Chinese Medicine, Fuzhou, Fujian 350122, China; ^4^National-Local Joint Engineering Research Center of Rehabilitation Medicine Technology, Fujian University of Traditional Chinese Medicine, Fuzhou, Fujian 350122, China; ^5^Fujian Key Laboratory of Rehabilitation Technology, Fuzhou, Fujian 350122, China; ^6^The Academy of Rehabilitation Industry, Fujian University of Traditional Chinese Medicine, Fuzhou, Fujian 350122, China; ^7^Key Laboratory of Orthopedics & Traumatology of Traditional Chinese Medicine and Exercise Rehabilitation, Ministry of Education, Fuzhou, Fujian 350122, China

## Abstract

**Background:**

To investigate the relationship between hypertension status, hippocampus/hippocampal subregion structural alteration, and cognitive performance in subjective cognitive decline (SCD).

**Methods:**

All participants were divided into two groups according to blood pressure status: SCD without hypertension and SCD with hypertension. The cognitive assessments and T1-MPRAGE brain MRI were performed to measure the cognitive function and the volume of the hippocampus and hippocampal subregions. Association and mediating/moderating effects were analyzed between the volume of hippocampus/hippocampal subregions and cognitive scores.

**Results:**

Compared to the SCD without hypertension, we found (1) increased reaction time (RT) of the Go/No go test, compatible test, and divided attention visual task and (2) decreased volume of the left whole hippocampal/left subiculum/left CA1/left presubiculum/left parasubiculum/left molecular layer HP/left GC-ML-DG/left HATA in SCD with hypertension. There was a significant negative association between the volume of the left GC-ML-DG and Go/No go test RT in SCD without hypertension. A significant moderating effect of hypertension status on the relationship between the volume of the left GC-ML-DG and Go/No go test RT was found.

**Conclusion:**

The results suggested that hypertension status affects inhibitory control function and visual divided attention which may be related to the reduction of hippocampus/hippocampal subregion volume in SCD. *Limitations*. The study has several limitations. First, this study does not include a healthy control group. In further studies, healthy controls may need to assess the interaction between hypertension status and disease status on cognitive function. Second, we defined the hypertension status using with or without hypertension disease. More detailed parameters of hypertension status need to be further studied. Third, our study was a small number of participants/single-center and cross-sectional study, which may hinder its generalization. A large-sample/multicenter, longitudinal study is helpful to comprehensively understand the relationship between hypertension status and cognitive function in SCD patients.

## 1. Introduction

Subjective cognitive decline (SCD) is characterized by self-reported memory or cognitive function decline, while no obvious cognitive dysfunction and the impairment of daily living ability in objective behavioral examination [1]. Although there is no objective cognitive impairment, compared to the healthy controls, the patients with SCD show a higher prevalence rate of memory, attention, motor, executive, and language function decline [2]. As a status between normal aging and mild cognitive impairment (MCI), SCD is considered to be one of the initial and first cognitive changes in the pathogenesis of Alzheimer's disease (AD) [3]. Compared to non-SCD older adults, SCD patients have a higher probability of progression to MCI or AD [4].

Hypertension, a highly prevalent disease all over the world (two-thirds of adults over 65 years old are hypertensive [5]), has been considered a well-established risk factor for cognitive decline in older adults [6]. A large-sample size (6732 participants) cross-sectional study from China suggested an age-dependent correlation between hypertension and cognition, and uncontrolled hypertension may be used as a predictor of cognitive decline in people over 75 years [7]. As the main target of the deleterious effects of hypertension on the brain, micro-cerebrovascular and macro-cerebrovascular lesions affect 40% to 50% of individuals with AD [8]. Hypertension affects multiple cognitive dimensions such as abstract reasoning, mental processing speed, and memory [9]. Uiterwijk et al. reported that SCD in patients with hypertension may relate to cognitive performance which suggested hypertension may be a risk factor in cognitive decline in SCD [10]. However, the neurobiological mechanism linking hypertension and cognitive function in SCD is still unclear, posing significant challenges for managing these increasing numbers of patients in the aging society.

There is potentially shared pathophysiology between hypertension and SCD. As a characteristic brain region of cognitive dysfunction, the hippocampus is considered to be one of the first affected regions in the pathological process of AD [11]. Compared to the healthy control, significant hippocampus atrophy was found in SCD [12]. An 8-year longitudinal study showed that the hypertension duration was independently associated with smaller hippocampus volume in older individuals [13]. The hypertension status may be a mediating variable between subjective memory complaint score and whole hippocampal volume in healthy elders [14] which suggests that the hypertension status may increase the cognitive decline via decreased hippocampus volume. Nevertheless, the participants of the study are healthy elders who are not diagnosed with SCD.

Furthermore, the hippocampal formation has complex anatomical organization and the subregions of the hippocampus have different properties and functional roles. The hippocampus is composed of the hippocampal tail, subiculum, dentate gyrus (DG), cornu ammonis (CA) 1–4, etc. [15]. For instance, the proteomes of areas CA1 and CA3 are largely different in the recognition of an object and the basal protein expression conditions [16]. In the progression of AD, specific anatomical areas of the hippocampus tend to atrophy [17]. In older adults with cognitive frailty, a significant volume decrease occurs in the bilateral presubiculum, the left parasubiculum, the hippocampal amygdala transition area (HATA), and the CA1 area [18], while compared to the MCI, the volume of the right molecular layer/right CA4 is significantly smaller and correlated with the cognitive performance (measured with MMSE) in AD [19, 20]. Although the above research showed difference alteration in the hippocampal subregions in the progression of AD, a comprehensive study on the structural characteristics of the hippocampal subregion networks which are affected by hypertension in SCD is lacking. Elucidating such hippocampal subregion alteration which is caused by hypertension in SCD could enhance our understanding of the basis for cognitive decline in SCD patients.

In the present study, we aimed to investigate the relationship between the hypertension status, hippocampus/hippocampal subregion structural alteration, and the cognitive scores in SCD. In this cross-sectional study, we collected T1-weighted MRI scans and the cognitive tests from SCD patients and compared hippocampal/hippocampal subregion structure and cognitive scores between SCD with hypertension and SCD without hypertension. In addition, we utilized moderating/mediating model to examine the moderating and mediating relationship between hypertension status, hippocampus subregions, and cognitive function in SCD. We hypothesized that the hypertension status may affect some special cognitive domains in patients with SCD and the altered cognitive function may associate with special hippocampal subregion structure of SCD.

## 2. Methods

### 2.1. Participants

Elderly subjects over 60 years old were recruited from July 2020 to December 2020 in Fuzhou City (Fujian province, China). After screening, a total of 120 SCD subjects were enrolled. The SCD was diagnosed using the SCD conceptual framework proposed by Jessen et al. in 2014 [1]. All participants were divided into two groups (SCD without hypertension and SCD with hypertension) according to their medical history and blood pressure (140 mmHg systolic blood pressure or 90 mmHg diastolic blood pressure) at the time of inclusion. This study was approved by the Medical Ethics Committee of the Affiliated Rehabilitation Hospital of Fujian University of Traditional Chinese Medicine, and all subjects gave informed consent and signed informed consent (Ethics Approval Number: 2020KY-011-01 and 2020KY-010-01).

The inclusion criteria were as follows: (1) meeting the diagnostic criteria for SCD [1]; (2) age between 60 and 75 years; (3) continued memory decline in self-perception compared to the previous normal state, independent of acute events; (4) no objective clinical impairment of MCI, Montreal Cognitive Assessment (MoCA) total score ≥ 26 points (if the education years ≤ 12 years, plus 1 point); (5) informed consent and voluntary participation.

The exclusion criteria include (1) uncontrolled hypertension; (2) history of alcohol and drug abuse; (3) severe anxiety and depression (Hamilton Depression Scale (HAMD) > 24 or Hamilton Anxiety Scale (HAMA) > 29); (4) cognitive decline caused by other reasons (such as nervous system disease like Parkinson' disease, Huntington's disease, mental disease, metabolic disease, poisoning, and infection); (5) metal implants (such as fixed metal dentures and pacemakers), taking drugs that affect brain imaging or other drugs that are not suitable for MRI scanning.

### 2.2. Cognitive Assessments

Global cognitive function was evaluated using the MoCA. The scale has a total score of 30 points, with higher scores indicating better overall cognitive function. Attentional performance was assessed by PSYTEST's Test of Attentional Performance (TAP, Version 2.3) [21]. Specifically, the divided attention subtest was administered. (Figure S1). The execute inhibit control function was assessed using the computerized “Go/No go” paradigms (Figure S2).

A compatible test was used to test the facilitation effect of SCD. In the test, arrows that are directed to the left or the right are presented on the left or the right of a fixation point. When the side of the stimulus in the visual field and the side of the responding hand (direction of the arrow) correspond, the subject should respond with the left or right hand irrespective of the side that the arrow presented.

### 2.3. MRI Data Acquisition

A 3.0 T Prisma scanner system (Siemens Medical Solutions, Erlangen, Germany) with a 64-channel head coil was used to collect the image data of subjects in this study. The T1-MPRAGE images were acquired with the following parameters: 15° flip angle (FA), 250 mm field of view (FOV), 160 slices, and 1 mm slice thickness. The subjects relaxed and opened their eyes without moving during scanning. We also collected an appropriate MRI scan (T2-weighted sequence) to check the serious vascular injuries such as stroke and brain tumor before study initiation. None of the eligible subjects included had obvious vascular lesions.

### 2.4. Brain Imaging Processing

All T1-weighted images were processed by the FreeSurfer software (version 7.1.0, https://www.freesurfer.net/) using default settings [22]. Before analyzing the image data, convert the DICOM data to NIFTI format using the MRI convert software. FreeSurfer automatic processing mainly includes head motion correction, nonbrain tissue removal, Talairach standard spatial registration, white matter segmentation, signal normalization, and topology correction/probabilistic atlas structure segmentation. According to the built-in atlas, the hippocampus can be divided into 12 subregions: hippocampal tail, subiculum, CA1, hippocampal fissure, presubiculum, parasubiculum, molecular layer of the HP, granule cell layer and molecular layer of the dentate gyrus (GC-ML-DG), CA3, CA4, fimbria, and hippocampal amygdala transition area (HATA). Total intracranial volume (TIV) was estimated as a covariate to reduce the effect of individual differences. The left and right hippocampal volumes were calculated separately in the present study.

### 2.5. Statistical Analysis

SPSS statistical software (IBM, Armonk, NY) was used for statistical analysis of the general demography data, behavioral data, and volumes of the hippocampus/hippocampal subregions, and *P* < 0.05 was considered statistically significant. The continuous variables conforming to a normal distribution were presented as the means ± standard deviations (SDs) and the independent sample *t*-test was used to compare characteristics between groups. When continuous variables did not conform to a normal distribution, the median (25–75th percentile) was presented and the Mann–Whitney *U* test was used to compare characteristics between groups. Categorical variables were described as frequencies, and the chi-square test was used to compare the characteristics between groups.

A general linear model or generalized linear model was used to compare the differences in the hippocampus and the volume of the hippocampal subregions between groups with the TIV as a covariate. The partial correlation was used to analyze the association between cognitive performances and hippocampal volumes with TIV as a covariate. All mediation and moderation analyses were performed using the PROCESS macro (http://processmacro.org/) [23] for SPSS. Model 4 was used to test the mediating effect of the hippocampal subregions on the relationship between hypertension status and cognitive function. Model 1 was used to test the moderating effect of hypertension status on the relationship between hippocampal subregions and cognitive function.

## 3. Results

### 3.1. Demographic Characteristics

No significant differences were found for age, gender, years of education, history of hyperlipidemia/Type II Diabetes Mellitus (T2DM) /cardiovascular disease (CVD), and the total scores of MoCA/HAMD/HAMA between the SCD without hypertension group and the SCD with hypertension group (Table 1).

### 3.2. The Comparisons of the Cognitive Function

The results showed that SCD with hypertension had significantly longer RTs in Go/No go test (*P* = 0.044) and compatible test (*P* = 0.016) compared to the SCD without hypertension. Compared to the SCD without hypertension group, the SCD with hypertension group showed significantly longer visual RTs in the divided attention test (*P* < 0.001). No other significant group difference was found in the cognitive performance (Table 2).

### 3.3. The Comparisons of Hippocampus and Hippocampal Subregion Volume

**Figure 1:**
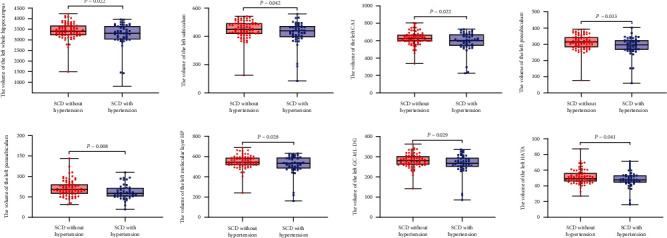
Comparison of the volume of the hippocampal subregions for participants. Compared to the SCD without hypertension, SCD with hypertension showed a significantly smaller volume of the whole left hippocampus, the left subiculum, the left CA1, the left presubiculum, the left parasubiculum, the left molecular layer HP, the left GC-ML-DG, and the left HATA.

We found that there was a significantly larger brain volume in the left whole hippocampal volume in the SCD without hypertension than in SCD with hypertension (*P* = 0.022). To further compare the difference in the left hippocampal subregion volume between the two groups, the volumes of the 12-left hippocampal subregions were analyzed. Seven hippocampal subregions had significant differences in volume between two groups. Specifically, the volumes of the left subiculum, the left CA1, the left presubiculum, the left parasubiculum, the left molecular layer HP, the left GC-ML-DG, and the left HATA in SCD with hypertension were significantly smaller than those in SCD without hypertension (Tables 3 and 4, Figure 1).

### 3.4. Correlation Analyses

**Figure 2:**
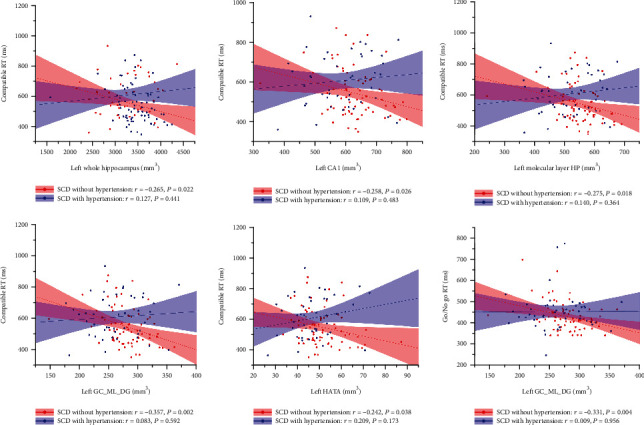
Correlation between the volume of the hippocampus/hippocampal subregions and cognitive scores in SCD. In the SCD without hypertension group, the volumes of the left whole hippocampus/CA1/molecular layer HP/GC-ML-DG/HATA were negatively associated with the compatible RTs, and the volumes of the left GC-ML-DG were negatively correlated to the Go/No go RT. No significant correlation between the volumes of hippocampal subregions and cognitive performance was found in SCD with hypertension.

Partial correlation analysis between cognitive scores and volumes of the hippocampal subregions showed significant negative correlation between the left whole hippocampus, the hippocampal subregions of the left CA1/molecular layer HP/GC-ML-DG/HATA with compatible RTs (*r* = −0.265, *P* = 0.022; *r* = −0.258, *P* = 0.026; *r* = −0.275, *P* = 0.018; *r* = −0.357, *P* = 0.002; *r* = −0.242, *P* = 0.038 separately), and volume of the left GC-ML-DG was negatively correlated with Go/No go RT in SCD without hypertension (*r* = −0.331, *P* = 0.004). No other significant correlation between the volume of hippocampal subregions and cognitive scores was found (Tables 5 and 6, Figure 2).

### 3.5. The Results of the Mediating and Moderating Effect Analyses

**Figure 3:**
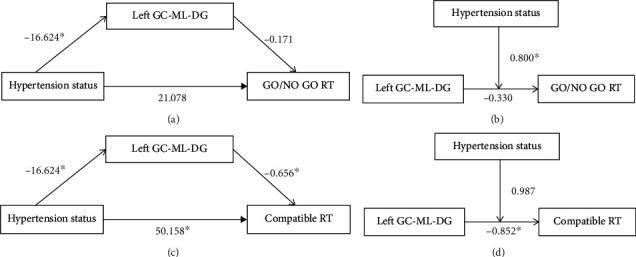
Model of mediating effects and moderating effects. (a) The mediating effect of the left GC-ML-DG between the hypertension status and the Go/No go RT was not significant. (b) The moderating effect of the hypertension status between the left GC-ML-DG and the Go/No go RT was significant. (c) The mediating effect of the left GC-ML-DG between the hypertension status and the compatible RT was not significant. (d) The moderating effect of the hypertension status between the left GC-ML-DG and the compatible RT was not significant (^∗^*P* < 0.05).

Since we found the volume of the left GC-ML-DG was negatively related with Go/No go RT/compatible RT in all subjects (Table 7), the mediating and moderating effect between hypertension status, the volume of the left GC-ML-DG, and Go/No go RT/compatible RT was analyzed. No significant mediating effects of the hippocampal subregions in the relationship between hypertension status and cognitive function were found. A significant moderating effect of hypertension status on the relationship between the left GC-ML-DG and Go/No go RT was found while the hypertension status did not significantly moderate the association between the left GC-ML-DG and compatible RT (Tables 8–11 and Figure 3).

## 4. Discussion

In the present study, we investigated the effects of hypertension status on the cognitive function and the hippocampus/hippocampal subregion volume in SCD. And the results suggested that hypertension status may affect inhibitory control/Stroop facilitation effects, attention, and volume of certain hippocampal subregions in SCD patients. In addition, we found that the hypertension status moderated the relationship between the volume of the left GC-ML-DG RT and Go/No go RT.

### 4.1. The Hypertension Status Affects the Cognitive Function in SCD

The Go/No go task is the main paradigm employed to examine response inhibition function [24]. The inhibition function is associated with the ability to successfully switch attention from the “Go” cue to the “No go” cue [25]. In the present study, we observed worse Go/No go task performance in SCD patients with hypertension, suggesting decreased inhibitory control in hypertensive SCD patients. In addition, we found an increased compatible RT in SCD with hypertension. Compatible RT is a classic indicator of the reversed Stroop effect. In the classical color-word Stoop task, there are two different processing, i.e., facilitation (compatible) and interference (incompatible) effects [26]. Due to the word meaning being processed automatically and interfered with the processing to color dimension [27], the RT incompatible > RT neutral and RT compatible < RT neutral [28]. The increased RT in the compatible condition at present indicated the attention automated processing may be compromised in SCD by the hypertension status. Furthermore, we found that SCD with hypertension had longer visual response times in divided attention. Recent studies have shown that the cognitive domains negatively affected by hypertension include abstract reasoning and/or executive function, memory, and mental processing speed [8, 29, 30]. The above findings of this study are similar to those of previous studies.

### 4.2. The Hypertension Status Affects Cognitive Performance Associated with the Specific Hippocampus/Hippocampal Subregion Volume in SCD

In this study, altered gray matter volume of the hippocampus in the left rather than in the right was found in SCD in different hypertension statuses which partly consisted and complemented previous studies and made the study of the hippocampus in patients with SCD more perfect from the structure MRI. Several studies have revealed the bilateral hippocampus asymmetry in the structure and function. Lister et al. found that the number and cell volume of neurons in the CA1 and CA2/3 regions of the right hippocampus are significantly less than those of the left [31]. Sakaguchiet et al. showed that the right hippocampal lesion impaired short-term memory performance and the left hippocampal lesion impaired the long-term memory performance. The right hippocampus has a facilitating role while the left hippocampus has a suppressing role for short-term memory performance. The left and right hippocampi of rodents may work in different mechanisms depending on the demand for short-term memory performance and long-term memory [32].

Our present study reported that the compatible RT was significantly associated with the volume of the left CA1/molecular-layer-HP and HATA of the left hippocampal subregions in SCD. A study from Crater showed that selective attention is involved in the facilitation effects of the Stroop task performance [33]. A recent study showed that compared to the normal control, there is significant hippocampal subregion atrophy over time including CA1 and molecular-layer-HP in the AD progression [34]. Attention and memory are complementary and synergistic in nature [35, 36]. Hippocampal memories can guide attention to previously inspected objects and thus facilitate quick object recognition. The study reported the CA1/molecular-layer-HP and HATA volume reduction in the disorders characterized by attention deficit such as Attention Deficit Hyperactivity Disorder (ADHD) [37]. The molecular layer and CA1 consist of interneuron synaptic connections and play an important role in regulating the activities within the hippocampus [38]. The reduction of the molecular layer and CA1 volume may indicate reduced interneuron connectivity between subfields of the hippocampus. The HATA is one of the main targets of the hippocampal-amygdala projection originating in the CA1 and located in the medial region of the hippocampus and closely connecting to the amygdala [39, 40]. The HATA plays a critical role in emotional learning and memory and social cognition [41]. These findings offer some insight into the mechanisms of hypertension status which contributes to the facilitation effects of the decline in SCD.

The subiculum is a primary receptive area for cortical projections to the hippocampus and a key hub in the formation and retrieval of episodic memory. The subiculum-containing detour loop is dedicated to meeting the recall processing in the rapid memory updating [42]. Neurofibrillary tangles (NFT), one of the major targets of AD hallmarks, target the subiculum of the hippocampus. Studies suggest that MCI patients with smaller hippocampus particularly in the CA1 and subiculum are at a higher risk of converting to AD [43]. In the present study, we found that the reduction volume in the left subiculum, left presubiculum, and left parasubiculum in the SCD with hypertension may suggest that SCD patients may be more likely to develop severe cognitive impairment if accompanied by hypertension, and good blood pressure control may reduce or reverse this possibility. More researches are needed in the future to confirm this hypothesis.

### 4.3. The Hypertension Status Moderated the Relationship between the Left GC-ML-DG and Response Inhibition Performance in SCD

In the present study, we observed worse Go/No go task performance which is significantly associated with the decreased hippocampus subregion volume in the left GC-ML-DG in SCD patients with hypertension. In addition, we found that hypertension status is a significant moderator variable between the left GC-ML-DG and the Go/No go task. These findings are partly consistent with a previous study that found the left DG is an early imaging biomarker in the damage of hypertension to the hippocampus impairment [44]. DG is the only main brain region of the neurogenesis in the hippocampal subregion [45] and inhibiting the hippocampal neurogenesis is one of the prominent manifestations of chronic hypertension [46]. Studies showed that the hippocampus is activated during the conflict processing resolution [47] and hippocampal beta power increases in Go/No go task [48]. SCD is an early stage in the processing of AD. Here, we showed that the DG volume may be an important contributor to the response inhibition function decline in SCD and controlling hypertension might be a potential target for delaying the cognitive decline.

### 4.4. Limitations

There are several limitations to the present study. First, as a preliminary study, the main purpose of this study is to investigate the effects of differences in hypertension status on the cognitive function in SCD; therefore, a healthy control group was not included in this study. A healthy control may need to assess the interaction effects of hypertension status (with or without hypertension) and disease status (healthy or SCD) on the cognitive function in further study. Second, at present, we defined the hypertension status using with or without hypertension disease. A recent study reported that the duration of hypertension/daytime mean systolic blood pressure was significantly associated with the performance of cognition [45, 49]. Further research with more detailed parameters of hypertension status is needed. Third, our present study is a small number of participants/single-center and cross-sectional study which may impede its generalizability. Nevertheless, this study supports the view that for SCD patients with hypertension, the cognitive function and hippocampus including the volume of the subregions may be affected. A large-sample size/multicenter and longitudinal study will help to comprehensively understand the relationship between hypertension status and cognitive functions in SCD.

## 5. Conclusion

In conclusion, our results demonstrate that the hypertension status influences the inhibition control function/Stroop facilitation effects and visual divided attention in patients with SCD. The volume of GC-ML-DG may affect the inhibition control function through the hypertension status in SCD. In addition, the combination of SCD and hypertension may cause some specific hippocampal subregions to atrophy. Our findings highlight new insights into the relationship between hypertension status and the cognitive function of SCD.

## Figures and Tables

**Table 1:** Demographics, medical history, and cognitive features of participants.

	SCD without hypertension (*n* = 75)	SCD with hypertension (*n* = 45)	*t*/*Z*/*x*^2^	*P*
Age (years)^a^	66.12 ± 4.80	66.31 ± 4.95	-0.209	0.835
Gender [*n* (%)]^b^			3.571	0.059
Male [*n* (%)]^b^	40 (53.3)	16 (35.6)		
Female [*n* (%)]^b^	35 (46.7)	29 (64.4)		
Education (years)^c^	12.00 (11.00-14.00)	12.00 (9.50-15.00)	-0.341	0.733
Hyperlipidemia [*n* (%)]^b^	2 (2.7)	4 (8.9)	2.292	0.130
T2DM [*n* (%)]^b^	11 (14.7)	11 (24.4)	1.796	0.180
CVD [*n* (%)]^b^	2 (2.7)	1 (2.2)	0.023	0.880
MoCA score^c^	27.00 (27.00-28.00)	28.00 (27.00-28.00)	-0.365	0.715
HAMD score^c^	2.00 (1.00-3.00)	2.00 (0.50-4.00)	-0.107	0.915
HAMA score^c^	3.00 (2.00-4.00)	2.00 (2.00-4.00)	-0.626	0.531

^a^Two-sample *t*-test was adopted, and mean ± SD was used for statistical description; ^b^chi-square test was adopted, and *n* (%) was used for statistical description; ^c^Mann–Whitney *U* test was adopted, and median (25-75th percentile) was used for statistical description. T2DM: type II diabetes mellitus; CVD: cardiovascular disease; MoCA: Montreal Cognitive Assessment; HAMD: Hamilton Depression Scale; HAMA: Hamilton Anxiety Scale.

**Table 2:** Performances of participants on the cognitive function.

	SCD without hypertension (*n* = 75)	SCD with hypertension (*n* = 45)	*t*/*Z*	*P*
Go/No go test				
RT (ms)^a^	416.00 (385.00-459.00)	449.00 (399.50-482.00)	-2.014	0.044
Valid responses^a^	20.00 (18.00-20.00)	19.00 (17.00-20.00)	-1.053	0.292
Compatibility test				
Compatible RT (ms)^a^	524.00 (453.00-611.00)	577.00 (498.00-732.00)	-2.404	0.016
Compatible valid responses^a^	30.00 (29.00-30.00)	30.00 (28.00-30.00)	-1.178	0.239
Divided attention				
Auditory RT (ms)^a^	753.00 (669.75-849.50)	748.00 (643.00-901.00)	-0.051	0.959
Auditory valid responses^a^	15.00 (13.00-16.00)	15.00 (11.00-16.00)	-0.456	0.648
Visual RT (ms)^b^	979.09 ± 161.16	1102.11 ± 194.50	-3.708	<0.001
Visual valid responses^a^	15.00 (14.00-16.00)	14.50 (13.00-16.00)	-1.058	0.290

^a^Mann–Whitney *U* test was adopted, and median (25–75th percentile) was used for statistical description; ^b^Two-sample *t*-test was adopted, and mean ± SD was used for statistical description.

**Table 3:** Comparisons of the hippocampal volume.

	SCD without hypertension (*n* = 75)	SCD with hypertension (*n* = 45)	Standardized *β*	*P*
Left whole hippocampus^a^	3408.86 (3231.65-3674.56)	3315.62 (3019.42-3654.47)	0.191	0.022
Right whole hippocampus^a^	3539.00 (3306.69-3781.17)	3428.98 (3172.27-3744.03)	0.096	0.241

^a^A generalized linear model was carried out with intracranial volume as the covariate, and the median (25–75th percentile) was used for statistical description. The unit of volume is cubic millimeters.

**Table 4:** Comparisons of the left hippocampal subregion volume.

	SCD without hypertension (*n* = 75)	SCD with hypertension (*n* = 45)	Standardized *β*	*P*
Left hippocampal tail^a^	576.06 (515.13-637.00)	559.12 (518.04-606.32)	0.110	0.178
Left subiculum^a^	450.35 (418.42-492.37)	439.17 (395.61-473.54)	0.173	0.042
Left CA1^a^	623.93 (594.36-668.53)	602.47 (541.52-671.78)	0.200	0.022
Left hippocampal fissure^a^	146.15 (130.26-167.15)	150.05 (135.18-171.57)	-0.012	0.891
Left presubiculum^a^	314.06 (281.57-343.76)	296.57 (266.64-324.49)	0.211	0.013
Left parasubiculum^a^	67.80 (57.59-80.18)	58.08 (50.86-71.81)	0.234	0.008
Left molecular layer HP^a^	544.74 (518.11-590.19)	534.61 (483.32-590.69)	0.187	0.028
Left GC-ML-DG^a^	280.37 (260.07-302.22)	267.24 (249.43-294.08)	0.185	0.029
Left CA3^a^	195.84 (177.49-216.31)	197.55 (179.54-214.10)	0.096	0.276
Left CA4^a^	243.77 (224.13-258.10)	233.05 (219.33-254.47)	0.162	0.056
Left fimbria^b^	71.94 ± 16.96	68.05 ± 20.33	0.098	0.274
Left HATA^a^	49.30 (45.64-56.29)	47.72 (44.02-53.27)	0.176	0.041

^a^A generalized linear model was carried out with intracranial volume as the covariate, median (25–75th percentile) was used for statistical description; ^b^a general linear model was carried out with intracranial volume as the covariate, and mean ± SD was used for statistical description. HATA: hippocampal amygdala transition area; GC-ML-DG: molecular layer of the dentate gyrus. The unit of volume is cubic millimeters.

**Table 5:** Correlation between the hippocampal subregion volume and cognitive scores in SCD without hypertension.

	Go/No go RT		Compatible RT		Divided attention visual RT	
	*r*	*P*	*r*	*P*	*r*	*P*
Left whole hippocampus	-0.208	0.076	-0.265	0.022	-0.024	0.839
Left subiculum	-0.061	0.608	-0.061	0.608	0.072	0.544
Left CA1	-0.172	0.144	-0.258	0.026	-0.073	0.540
Left presubiculum	-0.120	0.307	-0.095	0.422	-0.007	0.956
Left parasubiculum	0.058	0.625	-0.004	0.971	-0.102	0.391
Left molecular layer HP	-0.222	0.057	-0.275	0.018	-0.024	0.838
Left GC-ML-DG	-0.331	0.004	-0.357	0.002	-0.112	0.347
Left HATA	-0.151	0.199	-0.242	0.038	-0.059	0.619

Partial correlation between hippocampal subregion volume and cognitive scores in SCD without hypertension (*n* = 75) adjusting for intracranial volume. The unit of volume is cubic millimeters. HATA: hippocampal amygdala transition area; GC-ML-DG: molecular layer of the dentate gyrus.

**Table 6:** Correlation between the hippocampal subregion volume and cognitive scores in SCD with hypertension.

	Go/No go RT		Compatible RT		Divided attention visual RT	
	*r*	*P*	*r*	*P*	*r*	*P*
Left whole hippocampus	0.008	0.959	0.127	0.411	0.007	0.966
Left subiculum	0.063	0.686	0.152	0.325	-0.011	0.945
Left CA1	-0.023	0.882	0.109	0.483	0.063	0.687
Left presubiculum	0.032	0.839	0.191	0.215	0.061	0.696
Left parasubiculum	-0.199	0.194	0.085	0.585	0.183	0.241
Left molecular layer HP	0.040	0.797	0.140	0.364	0.018	0.907
Left GC-ML-DG	0.009	0.956	0.083	0.592	-0.042	0.789
Left HATA	-0.170	0.270	0.209	0.173	0.141	0.367

Partial correlation between hippocampal subregion volume and cognitive scores in SCD with hypertension (*n* = 45) adjusting for intracranial volume. The unit of volume is cubic millimeters. HATA: hippocampal amygdala transition area; GC-ML-DG: molecular layer of the dentate gyrus.

**Table 7:** Correlation between the hippocampal subregion volume and cognitive scores in all subjects.

	Go/No go RT		Compatible RT		Divided attention visual RT	
	*r*	*P*	*r*	*P*	*r*	*P*
Left whole hippocampus	-0.113	0.221	-0.130	0.157	-0.068	0.463
Left subiculum	0.007	0.941	-0.030	0.749	0.003	0.976
Left CA1	-0.093	0.312	-0.130	0.158	-0.080	0.389
Left presubiculum	-0.065	0.482	-0.023	0.805	-0.019	0.834
Left parasubiculum	-0.065	0.481	-0.034	0.716	-0.073	0.434
Left molecular layer HP	-0.091	0.322	-0.135	0.142	-0.071	0.445
Left GC-ML-DG	-0.191	0.037	-0.237	0.009	-0.158	0.088
Left HATA	-0.149	0.105	-0.117	0.201	-0.037	0.689

*N* = 120. The unit of volume is cubic millimeter. HATA: hippocampal amygdala transition area; GC-ML-DG: molecular layer of the dentate gyrus.

**Table 8:** Summary of the mediating effects of the left GC-ML-DG between the hypertension status and the Go/No go RT.

	Effect	SE	LL 95% CI	UL 95% CI
Total effect	23.924	14.248	-4.291	52.140
Direct effect	14.515	1.452	-7.667	49.823
Indirect effect	2.846	2.974	-3.254	9.001

Bootstrap size = 5000; LL: lower limit; CI: confidence interval; UL: upper limit; SE: standard error.

**Table 9:** Summary of the mediating effects of the left GC-ML-DG between the hypertension status and the compatible RT.

	Effect	SE	LL 95% CI	UL 95% CI
Total effect	61.067	23.773	13.990	108.144
Direct effect	50.158	23.749	0.037	3.124
Indirect effect	10.909	7.914	-0.570	29.581

Bootstrap size = 5000; LL: lower limit; CI: confidence interval; UL: upper limit; SE: standard error.

**Table 10:** Moderating effect of the hypertension status between the left GC-ML-DG and the Go/No go RT.

	Effect	SE	*t*	*P*
Constant	441.142	6.881	64.112	<0.001
Left GC-ML-DG (*x*)	-0.330	0.177	-1.866	0.045
Hypertension status (*w*)	21.771	14.227	1.530	0.129
Left GC‐ML‐DG^∗^ hypertension status (*x*^∗^*w*)	0.800	0.331	2.414	0.017

Independent variable (*x*) = left GC-ML-DG; dependent variable (*y*) = Go/No go RT; moderator (*w*) = hypertension status.

**Table 11:** Moderating effect of the hypertension status between the left GC-ML-DG and the compatible RT.

	Effect	SE	*t*	*P*
Constant	572.613	11.380	50.318	<0.001
Left GC-ML-DG (*x*)	-0.852	0.292	-2.916	0.004
Hypertension status (*w*)	51.012	23.529	2.168	0.032
Left GC‐ML‐DG^∗^ hypertension status (*x*^∗^*w*)	0.987	0.548	1.801	0.074

Independent variable (*x*) = left GC-ML-DG; dependent variable (*y*) = compatible RT; moderator (*w*) = hypertension status.

## Data Availability

The data used to support the findings of this study are available from the corresponding author upon request.

## Supplementary Materials

Supplementary Materials In the test of divided attention, a visual and an auditory task must be processed in parallel. Visual task: a quadratic field of dots (4 × 4) appears in the central area of the screen in which a varying number of crosses appear simultaneously. The subject presses the reaction key as quickly as possible when four crosses appear in neighboring positions forming a small square. Auditory task: a high and low tone is emitted alternately according to the synchronous rhythm of the changing position of the crosses. When the high or low tones are emitted twice in succession, the subject must press the reaction key as quickly as possible (Figure S1). In the “Go/No go” test, an upright (“+”) and a diagonal cross (“×”) are presented in an alternating sequence on the screen. The subjects react as quickly as possible whenever “×” appears; no reaction is required when the “+” cross appears (Figure S2).
