# Supplementary material for: Hypertension Status Moderated the Relationship between the Hippocampal Subregion of the Left GC-ML-DG and Cognitive Performance in Subjective Cognitive Decline
Source: Dis Markers. 2022 Oct 15;2022:7938001. doi: 10.1155/2022/7938001 (PMC9588336; doi:10.1155/2022/7938001)
Supplement: Supplementary Materials — In the test of divided attention, a visual and an auditory task must be processed in parallel. Visual task: a quadratic field of dots (4 × 4) appears in the central area of the screen in which a varying number of crosses appear simultaneously. The subject presses the reaction key as quickly as possible when four crosses appear in neighboring positions forming a small square. Auditory task: a high and low tone is emitted alternately according to the synchronous rhythm of the changing position of the crosses. When the high or low tones are emitted twice in succession, the subject must press the reaction key as quickly as possible (Figure S1). In the “Go/No go” test, an upright (“+”) and a diagonal cross (“×”) are presented in an alternating sequence on the screen. The subjects react as quickly as possible whenever “×” appears; no reaction is required when the “+” cross appears (Figure S2). [file 7938001.f1.docx]

**Supplementary Materials**

**
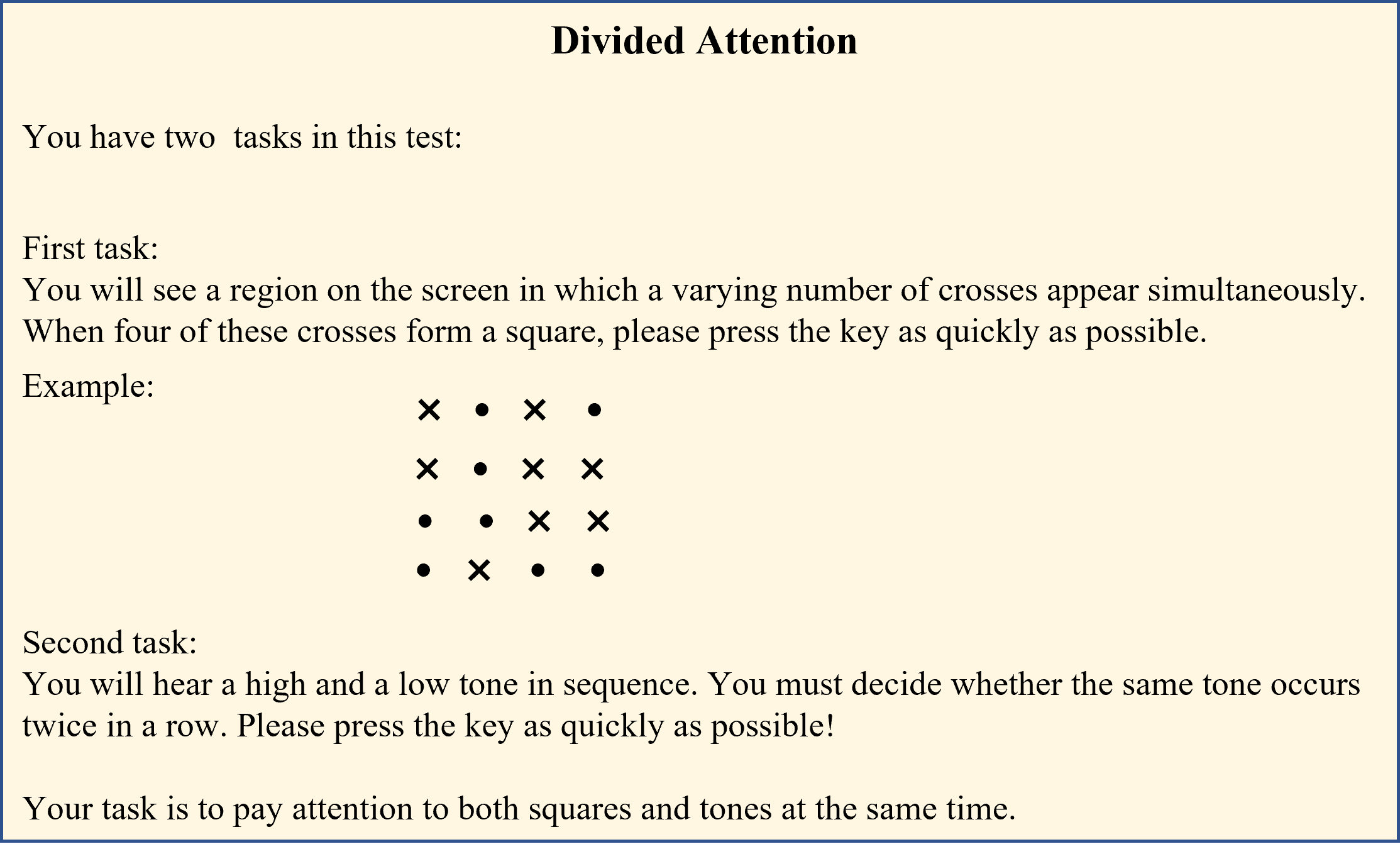
**

**Figure S1: Instruction for** **divided attention test**

In this test, a visual and an auditory task must be processed in parallel. Two forms of this test may be administered. Visual task: a quadratic field of dots (4 × 4) appears in the central area of the screen, together indicating 16 positions at which between 6 and 8 small crosses may appear during the test in a predetermined rhythm. The subject has to press the reaction key as quickly as possible when 4 crosses appear in neighbouring positions such that they together form the corners of a small square. Auditory task: a high and low tone is emitted alternately according to the synchronous rhythm of the changing position of the crosses. From time to time, the high or low tones are emitted twice in succession. The subject must also in this case press the (identical) reaction key as quickly as possible.

**
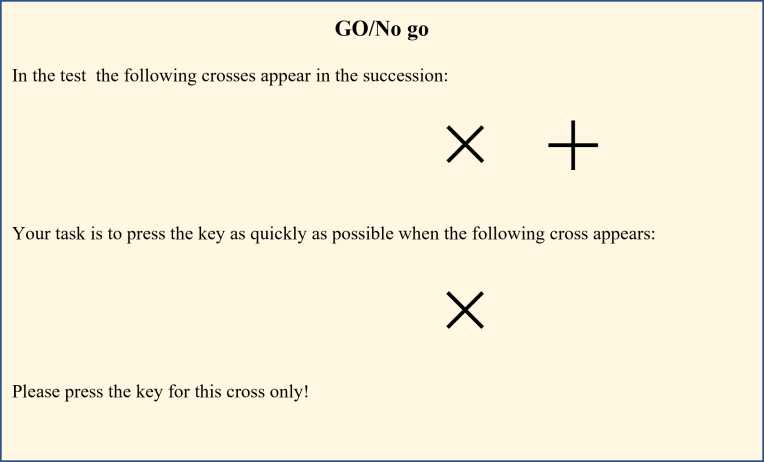
**

**Figure S2: Instruction for** **Go/No go test**

In this test, the subject has to react as quickly as possible with a key press whenever the diagonal cross appears (“×”); no reaction is required when the up-right cross appears (“+”).
